# Growth and Specialized Growth Charts of Children with Congenital Hypothyroidism Detected by Neonatal Screening in Isfahan, Iran

**DOI:** 10.1155/2013/463939

**Published:** 2013-02-07

**Authors:** Awat Feizi, Mahin Hashemipour, Silva Hovsepian, Zeynab Amirkhani, Roya Kelishadi, Maryam Yazdi, Kamal Heydari, Ali Sajadi, Masoud Amini

**Affiliations:** ^1^Department of Biostatistics and Epidemiology, School of Health and Endocrinology and Metabolism Research Center, Isfahan University of Medical Sciences, Isfahan, Iran; ^2^Endocrinology and Metabolism Research Center and Child Growth and Development Research Center, Isfahan University of Medical Sciences, Isfahan 8174673837, Iran; ^3^Isfahan University of Medical Sciences, Isfahan, Iran; ^4^Child Growth and Development Research Center, Isfahan University of Medical sciences, Isfahan, Iran; ^5^Department of Biostatistics and Epidemiology, School of Health, Isfahan University of Medical Sciences, Isfahan, Iran; ^6^Department of Social Dentistry, Isfahan Province Health Center, Isfahan, Iran; ^7^Isfahan Province Health Center, Isfahan, Iran; ^8^Endocrinology and Metabolism Research Center, Isfahan University of Medical Sciences, Isfahan, Iran

## Abstract

*Objectives.* The aim of the current study was to investigate the growth status of CH, generate specialized growth charts of CH infants, and compare them with their counterparts of regional normal infants. *Methods.* In this prospective cohort study, 760 (345 girls and 415 boys) neonates born in 2002–2009 diagnosed by neonatal CH screening program in Isfahan were followed up from the time of diagnosis. 552 healthy children were recruited as a control group. The empirical 3rd, 15th, 50th, 85th, and 97th percentiles for height, weight, and head circumference of both sexes were determined and compared with their counterpart values of the control group. The relative frequency of patients with impaired growth for each studied variable was determined. Also, specialized growth charts of CH patients were generated. *Results.* The percentiles of weight, height, and head circumference of studied patients are significantly different from regional healthy children (*P* < 0.001). The relative frequency of impaired head circumference was decreased to less than 3% at the 3rd year of age and for height it reached gradually 3% and 9% at the 5th year of age for boys and girls, respectively (*P* < 0.05); however for weight still it was statistically more than 3% in both sexes. *Conclusion.* CH patients had impaired growth development which was improved during follow up, but the catch-up time was earlier for head circumference and later for weight.

## 1. Introduction

Thyroid hormones are essential for appropriate growth and development during fetal life and neonatal period [1]. Congenital hypothyroidism (CH), the most common neonatal endocrine and metabolic disorder, is considered as the preventable cause of mental and growth retardation [2].

Congenital hypothyroidism (CH) is the most common congenital endocrine disorder, affecting 1 in 3000 to 4000 newborns. The prevalence varies depending on the race/ethnicity and the method of screening [2–7]. 

Although screening programs ensure early treatment, developmental problems in relation to physical and mental outcomes are still reported in follow-up studies in developing and even in developed nations [8–10].

CH screening in Isfahan, one of the biggest central provinces in Iran, has been started in 2002 as a pilot study which continued till 2005 and because of high prevalence of the disorder, that is, 1 : 370 in 2002 [7] and 1 : 748 in 2009 [11], it was adapted as a nationwide screening program in Iran and is continuing till now [11]. Many studies have already been performed and published regarding the high prevalence and its environmental, genetic, and ethnic determinants and the outcome of CH treatment [7, 12–16]. However, growth evaluation of CH patients who were diagnosed and referred for treatment and followup has not been studied in this region. 

Several studies have reported controversial findings about the growth of CH in hypothyroid neonates, some indicated that early treatment had resulted in normal growth of the mentioned patients, whereas others had not indicated the same [17–19]. However, it seems that the growth catch-up of the CH patients depends on many factors such as age of treatment, dosage of treatment, and the severity of the disorder [20]. Determining the growth status of CH patients in comparison with normal infants would help us to improve our screening and treatment strategies to achieve the best result in this field. So, considering that there is no study in this field in Iran and particularly the importance of the issue because of high prevalence of CH in Isfahan, the aim of the current study was to evaluate the growth status of CH patients by computing different empirical percentiles of their height, weight, and head circumference and comparing them with Iranian normal infants. Furthermore, as a novel work for the first time over the world, the specialized growth curves for these patients were generated and compared with their counterparts in normal children.

## 2. Methods

In this prospective cohort study, 760 (345 girls (45%) and 415 boys (55%)) congenitally hypothyroid neonates diagnosed and referred to Isfahan Endocrinology and Metabolism Research Center for treatment and followup during CH screening program (2002–2009) were enrolled. CH screening program was initiated in 2002 and continued till 2005, when nationwide CH screening program was implemented. During the screening program, a pediatric endocrinologist and collaborating general practitioners evaluated the laboratory results and the neonates who needed to be recalled were determined. The need for recall was based on the measured T4 and TSH levels. Neonates with a TSH >20 mIU/L or a T4 <6.5 mg/dL on the third–seventh days of life were recalled. Beyond day 7, neonates were recalled if their measured TSH was >10 mIU/L or their T4 was <6.5 mg/dL.

Based on the results of the secondary measurements (between days 7 and 28), neonates were considered hypothyroid if their T4 was <6.5 mg/dL and their TSH was >10 mIU/L [21–23]. In both preterm and full-term neonates whose T4 measurements were low according to their weight [24], further tests including T3 resin uptake (T3RU) and free T4 index (FTI) were performed, and treatment was started after confirmation of secondary hypothyroidism or TSH 10 mIU/L. More details about diagnosis criteria over the screening program can be found elsewhere [11].

Hypothyroid neonates underwent treatment within 15 to 30 days at a dose of 10–15 *μ*g/kg/day after the diagnosis was confirmed, with the monitoring of TSH and T4 every 1-2 months during the first year of life and every 1–3 months during the second and third years. If the child remains hypothyroid at 3 years of age, thyroid hormone replacement and medical monitoring are usually required for life.

During each follow-up time point, in addition to evaluating thyroid function tests, physical development of CH patients was evaluated through measuring weight, height, and head circumference. Data of the patients from diagnosis till the last followup was recorded in their profile information (i.e., 3-4 record for the first year and 2-3 record after 2 years of age). Weight and height were recorded till age ≤5 years; however head circumference was measured till 3 years of age. The children's height was measured in supine position before walking and then in standing position. Weight was measured with a precision of 10 g, and height and head circumference of 10 mm. At each followup all these parameters were measured in the same scale. CH patients with no data or obvious errors in their demographic information and anthropometric measurements were excluded. Those with concomitant disease or complications such as prematurity, IUGR, genetic disorders, and congenital malformations were excluded also. Finally, of 924 CH patients, 760 were enrolled. For comparison purposes, information recorded in profile of 552 (270 (48.9%) girls and 282 (51.1%) boys) healthy children, as control group, in health centers affiliated to Isfahan University of Medical Science was used. Sample selection process for normal children was done using multistage cluster sampling. The ethics committee of Isfahan University of Medical Sciences approved the study.

### 2.1. Statistical Analysis

Obtained data was analyzed using SPSS 16 software. Statistical methods included obtaining the empirical percentiles including 3rd, 15th, 50th, 85th, and 97th for each variable of both sexes and comparing them with counterpart values of Iranian normal infants as well as the values reported by WHO. Differences in proportions between CH patients and normal infants in mentioned quantiles were evaluated using chi-square test in each age and sex group. Also, 3rd percentile of studied variables in normal infants in each follow-up points was considered as reference values and the proportion of CH patients with values less than this cut point, as growth retardation, were calculated and tested using one-sample proportion statistical test considering 3% as the test value.

### 2.2. Specialized Growth Chart for CH Patients

As an important objective of the current study, sex-specific growth charts of weight, height, and head circumference of CH patients were created using additive models for quantile regression. This model also was used for generating growth charts of normal children. Through these growth curves, development of CH patients can easily be compared with normal infants in follow-up period graphically. R free statistical software version 2.13.1. was used for generating the growth charts. 

## 3. Results

760 CH patients studied in this study consisted of 345 girls (45%) and 415 boys (55%), of these patients 65, 96, 84, 78, 113, 123, 111, and 90 were born in 2002, 2003, 2004, 2005, 2006, 2007, 2008, and 2009, respectively.

As descriptive findings, the 3rd, 15th, 50th, 85th, and 97th empirical percentiles of weight, height, and head circumference for studied samples were calculated. Tables 1, 2, and 3 provide the values of these quantities and their counterpart values of regional normal children and WHO. Chi-square statistical test for goodness of fit showed that the calculated percentiles of study sample for all studied variables are statistically different from the corresponding values of normal children and WHO; in which for both sexes and in each age group the obtained percentiles from studied participants are less than Iranian normal as well as WHO counterpart values; the differences were statistically significant for weight and height at *P* < 0.001 and for head circumference at *P* < 0.05. 

**Figure 1:**
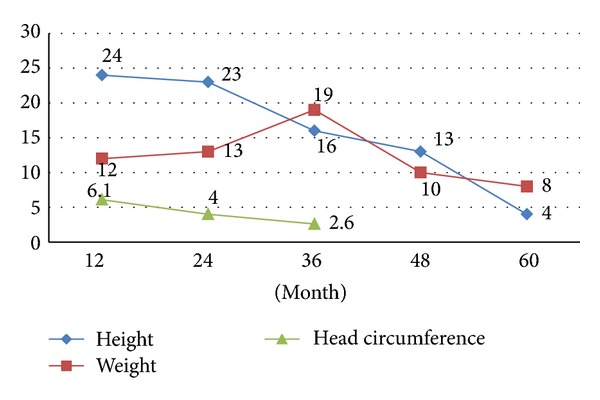
The percent of CH patients (girls) with impaired growth in terms of studied variables over follow-up period.

**Figure 2:**
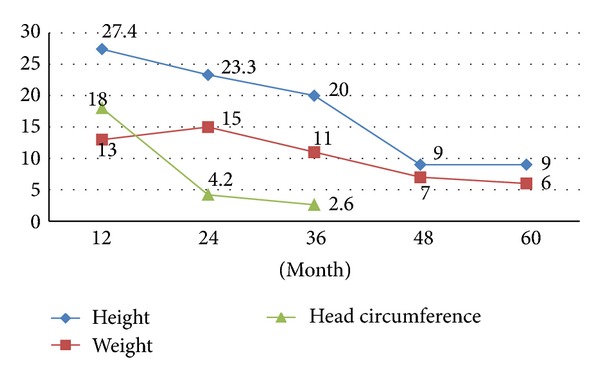
The percent of CH patients (boys) with impaired growth in terms of studied variables over the follow-up period.

The relative frequency percents for boys and girls CH patients with impaired growth (based on comparisons with 3th of Iranian normal children) for all studied variables over the follow-up period are presented in Figures 1 and 2. The percent of patients with growth retardation in terms of each studied variable was significantly higher than 3% of the total studied population, which means that more than 3% of studied CH patients had the impaired growth (*P* < 0.05). The results indicated that the relative frequency of development retardation for height of both sexes in CH patients was decreased significantly during treatment period in a way that it gradually reached 3% and 9% at 5 years of age in girls and boys, respectively (*P* < 0.05 for girls and *P* > 0.05 for boys, Figures 1 and 2). It means that the percent of girls with impaired growth is not more than 3% but for males it is still more than 3% and this difference is statistically significant. The relative frequency of patients with impaired growth in terms of head circumference reached <3% at 3 years of age in both sexes (*P* < 0.05) (Figures 1 and 2).

Regarding weight, the findings showed decreasing trend; but relative frequency of growth retardation at the end of five-year followup still was statistically more than 3% in both sexes so that the decrease was not statistically significant (*P* > 0.1).

**Figure 3:**
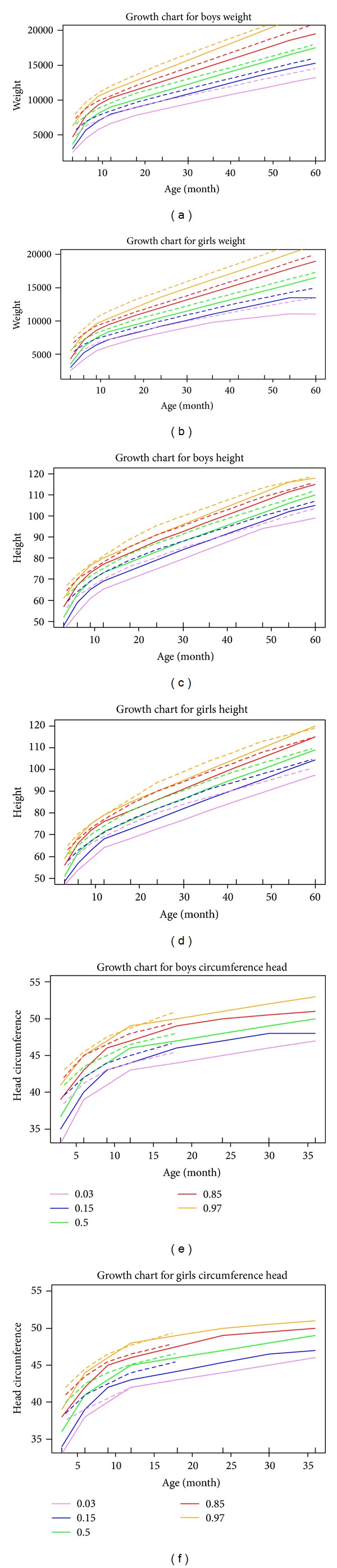
Comparison between Iranian normal children (dashed curves) and CH patients (solid curves) in terms of percentiles (growth charts) of weight, height, and head circumference for age for both sexes.

We generated growth charts for boys and girls from 0 to 5 years old for weight and height while for head circumference from 0 to 3 for CH patients and 0 to 1.5 years old for normal children (it should be noted that the registered data regarding head circumference for normal children was available from 0 to 18 months). Figures 3(a) to 3(d) depict the comparative growth charts of weight and height for age in boys and girls from birth to 60 months; also Figures 3(e) and 3(f) represent the growth charts of head circumference for age in normal boys and girls from birth to 18 months while to 36 months for CH patients. In Figures 3(a) to 3(f), the dash and solid curves represent the development of normal and CH patients, respectively. In each chart, a set of percentiles including 3th, 15th, 50th, 85th, and 97th were calculated. As can be seen from these charts, as age is increasing, the growth status of CH patients tends to be more similar with normal people particularly over the upper percentiles. 

## 4. Discussion

In the current cohort study the growth of CH patients diagnosed during the CH screening program and followed up from 2002 to 2009 was investigated. The findings of this study indicated that totally the obtained percentiles of each studied variable for CH patients were different significantly from their counterpart values of the healthy children as well as of WHO but the difference becomes less along with growing age. Investigating the relative frequency of CH patients with impaired growth in terms of each variable indicated that more than 3% of CH patients had impaired growth which was decreased by increasing age for height and head circumference more notable and had a steeper decreasing trend for weight.

 Though according to recent studies both in Iran and other countries the WHO Child Growth Standards considered it as an appropriate tool for assessing the growth of infants and young children and it can be used properly to assess the well-being of children everywhere, regardless of ethnicity, socioeconomic status, and type of feeding but it is more appropriate to compare the percentiles with regional ones [18, 25–27]. Accordingly, considering that there were not any reference percentile curves for studied variables for regional children, in the current study for comparative purposes the empirical percentiles and growth charts for healthy children based on a sufficient sample size and powerful statistical method were generated.

The results of the current study indicated that though CH patients had impaired growth compared with normal ones, but due to treatment this growth impairment decreased during followup and increasing age. Our results were in accordance with the results of Grant et al.'s in London which indicated that by the age of 3-4 years stature became normal in children with early treated congenital hypothyroidism [20]. Chiesa and colleagues in their study on growth followup of 100 CH patients before and during adequate treatment up to 5 years of age have showed that thyroid hormone replacement in CH as late as 24 months corrects the short stature and delayed bone age by age 5 years [28]. Also Kik and Noczyńska in their study concluded that physical development of children in infancy was in normal range; for instance, mean (SD_S_) for body length in all age groups was in the range ±1 SD_S_ of healthy population [27]. However several data have reported that the majority of CH patients detected by neonatal screening had normal growth, in general, from the first weeks of life [28–31], as reported by Sato et al. in Japan. They reported that only CH patients with complications had impaired growth [31]. In a recent study in China, Sun et al. have investigated the height, weight, and body mass index (BMI) of CH patients in comparison with normal group of children that was diagnosed and followed up during a 12-year period and concluded that there was no significant difference between mentioned groups and children with CH that has been identified by newborn screening and early treatment [32]. In another study in Spain, in 2010, the physical and psychological development of CH patients was evaluated and according to their results the physical development of CH children was not different from that of the healthy population of Barcelona and the most delayed normalization was in relation with their psychological development [33].

Our study showed that the height in more than 3% of CH patients of both sexes was impaired which decreased significantly during followup and reached 3% and 9% in females and males, respectively, at 5 years of age. There are controversial reports in this field; some indicated that there was no difference in height of CH patients compared with normal children but other reported delayed height growth which improved during treatment which is also dependent on the dose of levothyroxine and the timing of treatment initiation [33–35].

Morin et al. in Argentina studied linear growth of CH patients and concluded that they reached normal height value at 3 years of age [35]. Moschini et al. reported that CH patient sreached normal height at 6 years of age if treatment initiation would be in 33 days after birth [29]. Normal height at 6 years of age was also reported by Siragusa et al. [36]. Heyerdahl and colleagues in their study on linear growth of early treated CH patients reported that, in comparison with reference children, children with hypothyroidism had reduced growth from 6 to 12 months and increased growth after 12 months of age. They concluded that thyroid hormones during the first months of life are essential for normal growth of children. Their results indicated that both the infancy and the childhood components of growth are thyroid hormone dependent [37].

The current study indicated that over the entire follow-up period more than 3% of CH patients of both sexes had impaired growth and a decreasing trend in this proportion was observed though it was not statistically significant. Sato et al. have reported normal weight for CH patients [31], but others have reported that CH patients were heavier than normal children even during childhood and adolescence period, in contrast with our findings [38]. It is suggested that higher weight in CH patients is prominently related to inappropriate treatment such as undertreatment condition as a result of parent-child or physician-child relationship and is not related to factors related to linear growth as mentioned by other studies [39]. 

Head circumference of studied participants had reached ≤3% in the third year of age and was lower than normal population. Previous studies showed that CH patients had larger head circumference especially those with thyroid agenesis, which reached normal value by treatment [38, 39]. In contrast to other studies [38], in the current survey, the head circumference was lower than normal value, and it may be due to that the majority of CH patients in Isfahan were dyshormonogenetic ones [40], or it may be due to proper and early treatment. However it needs more studies for more accurate conclusion.

In conclusion, the descriptive findings of the present research indicated that in treated CH patients the growth in physical development had delay which improved during followup in which it tended toward normal status; the catch-up time was earlier for head circumference, after that for height, and later for weight. However the findings of our study showed that early treatment of congenital hypothyroidism leads to improving outcomes; we could not deny the fact that some patients were treated insufficiently because of factors related to both parents and the physicians, Although the findings of our study showed that the early treatment of congenital hypothyroidism leads to improving outcomes; we could not deny the fact that some patients treated insufficiently because of factors related to both parents and the effects of some important factor such as disease severity, timing of onset, age at starting therapy that all influence the results should be considered. Currently a comprehensive research setting using advanced statistical modeling for longitudinal data about the determinants of growth development in these patients in Isfahan's Endocrinology and Metabolism Research Center is undertaken. 

## Figures and Tables

**Table 1:** Percentiles of Weight (gr) for age (months) for CH patients, Iranian normal girls and boys (highlighted in italic) and WHO corresponding values (presented in parentheses).

			Percentiles									
Age	Gender		3		15		50		85		97	
(months)	(*N*)		(WHO)		(WHO)		(WHO)		(WHO)		(WHO)	
	Female	Male	Female	Male	Female	Male	Female	Male	Female	Male	Female	Male
3	308	371	2550 (4600)	2450 (5100)	3000 (5100)	3000 (5600)	3475 (5800)	3600 (6400)	4300 (6700)	4750 (7200)	5510 (7400)	6250 (7900)
Iran-regional *			*4460 *	*4450 *	*50500 *	*5400 *	*56250 *	*6150 *	*63500 *	*6950 *	*6980 *	*7650 *
6	385	343	4000 (5800)	4460 (6400)	5150 (6400)	5700 (7100)	6100 (7300)	6800 (7900)	7100 (8300)	7800 (8900)	8000 (9300)	8950 (9700)
*Iran-regional *			*5755 *	*6250 *	*64500 *	*6900 *	*72000 *	*7800 *	*8100 *	*8850 *	*9265 *	*9800 *
9	367	315	5500 (6600)	5800 (7200)	6400 (7300)	7000 (7900)	7500 (8200)	8100 (8900)	8650 (9300)	9350 (10000)	9650 (10400)	10600 (10900)
*Iran-regional *			*6700 *	*7100 *	*73000 *	*7800 *	*82000 *	*8800 *	*9300 *	*9900 *	*10551 *	*11000 *
12	232	280	6300 (7100)	6650 (7800)	7200 (7900)	8000 (8600)	8400 (8900)	9100 (9600)	9550 (10300)	10500 (10800)	10500 (11300)	11500 (11800)
*Iran-regional *			*7100 *	*7850 *	*7900 *	*8450 *	*8750 *	*9600 *	*9890 *	*10600 *	*11680 *	*12000 *
18	248	295	7350 (8300)	7850 (8900)	8200 (9000)	8800 (9700)	9400 (10200)	10000 (10900)	10900 (11600)	11500 (12300)	12000 (13000)	12800 (13500)
*Iran-regional *			*8300 *	*9000 *	*9000 *	*9700 *	*10250 *	*11000 *	*11400 *	*12200 *	*13700 *	*13800 *
24	213	263	8100 (9300)	8560 (9800)	9350 (10100)	10000 (10800)	10500 (11500)	11300 (12300)	12200 (13100)	13000 (13700)	14100 (14000)	14400 (15100)
*Iran-regional *			*9000 *	*9850 *	*10000 *	*10600 *	*11200 *	*12000 *	*12800 *	*13500 *	*14720 *	*15700 *
30	158	233	8880 (10100)	9600 (10700)	10000 (11300)	10800 (11800)	11100 (12700)	12000 (13300)	13000 (14500)	13800 (15000)	14600 (16200)	15500 (16600)
*Iran-regional *			*10000 *	*10600 *	*10940 *	*11550 *	*12250 *	*13000 *	*13770 *	*14500 *	*15350 *	*16600 *
36	158	192	10000 (11000)	10000 (11400)	11000 (12100)	12000 (12700)	12500 (13900)	13500 (15900)	14500 (15900)	15000 (16300)	16300 (17800)	16500 (18000)
*Iran-regional *			*10500 *	*11000 *	*11750 *	*12500 *	*13200 *	*14000 *	*15000 *	*15700 *	*17000 *	*18000 *
42	94	148	10000 (11800)	10235 (12200)	11500 (13100)	12600 (13500)	13500 (15000)	14500 (13500)	15500 (17300)	16000 (17500)	17150 (19400)	18530 (19500)
*Iran-regional *			*11200 *	*12000 *	*12750 *	*13250 *	*14250 *	*15000 *	*16250 *	*17000 *	*18650 *	*18750 *
48	80	117	10600 (12500)	11954 (12900)	12500 (14000)	14000 (14300)	14500 (16100)	15200 (16300)	16500 (18600)	18000 (18700)	19500 (20900)	20000 (21100)
*Iran-regional *			*12300 *	*13000 *	*13500 *	*14250 *	*15300 *	*16000 *	*17850 *	*18500 *	*21000 *	*21300 *
54	53	59	11300 (13200)	10900 (13600)	13800 (14800)	13800 (15200)	15500 (17200)	16500 (17300)	18500 (20000)	20000 (19900)	20000 (22300)	21700 (22800)
*Iran-regional *			*12600 *	*13800 *	*14250 *	*15400 *	*16250 *	*17400 *	*18800 *	*20000 *	*21500 *	*22000 *
60	34	48	11200 (14000)	13650 (14300)	15000 (15700)	15050 (16000)	17000 (18200)	17500 (18300)	20000 (21100)	19500 (21300)	22000 (23800)	26600 (24400)
*Iran-regional *			*13750 *	*14500 *	*15000 *	*16000 *	*17500 *	*18500 *	*20000 *	*21000 *	*23000 *	*24200 *

*Sample sizes for Iranian normal boys and girls, in all age groups, were 282 and 270 respectively.

**Table 2:** Percentiles of Height (cm) for age (months) for CH patients, Iranian normal girls and boys (highlighted in italic) and WHO corresponding values (presented in parentheses).

			Percentiles									
Age	Gender		3		15		50		85		97	
(months)	(*N*)		(WHO)		(WHO)		(WHO)		(WHO)		(WHO)	
	Female	Male	Female	Male	Female	Male	Female	Male	Female	Male	Female	Male
3	301	364	47 (55.8)	45.9 (57.6)	48.9 (57.6)	48 (59.3)	51 (59.8)	52 (61.4)	56 (62)	57 (63.5)	59 (63.8)	61.05 (65.3)
Iran-regional *			*54 *	*54.6 *	*56.7 *	*58 *	*59.5 *	*60.5 *	*61.5 *	*63 *	*63.2 *	*65.5 *
6	281	341	54 (61.5)	54 (63.6)	57 (63.4)	59 (65.4)	62 (65.7)	63 (67.6)	66 (68.1)	67 (69.8)	69.54 (70)	70 (71.6)
*Iran-regional *			*61 *	*62 *	*63 *	*64 *	*65 *	*67 *	*68 *	*70 *	*71 *	*72.3 *
9	266	309	59 (65.6)	61 (67.7)	63 (76.6)	65 (69.6)	67 (70.1)	69 (72)	72 (72.6)	73 (74.3)	75 (74.7)	76.8 (76.2)
*Iran-regional *			*66 *	*66 *	*67 *	*69 *	*70 *	*71.5 *	*73 *	*74 *	*75.2 *	*77 *
12	224	276	64 (69.2)	66 (71.3)	68 (71.3)	69 (73.3)	72 (74)	73 (75.7)	76.2 (76.7)	77 (78.2)	79 (80.2)	80 (83.7)
*Iran-regional *			*69 *	*70 *	*71 *	*73 *	*74 *	*75 *	*77 *	*78.1 *	*80 *	*81 *
18	246	289	68.4 (75.2)	70 (77.2)	72 (77.7)	73.5 (79.5)	76 (80.7)	78 (82.3)	80.9 (83.7)	82 (85.1)	85 (82.6)	85 (87.9)
*Iran-regional *			*75 *	*76 *	*77 *	*79 *	*81 *	*82 *	*84 *	*85.5 *	*86.1 *	*89 *
24	205	261	72 (80.3)	74 (82.1)	78 (83.1)	79 (84.6)	82 (86.4)	84 (87.7)	86.1 (89.8)	88 (91)	90 (92.5)	92 (93.6)
*Iran-regional *			*79.8 *	*80.4 *	*82 *	*84 *	*86 *	*87.5 *	*90 *	*91 *	*94.2 *	*95.6 *
30	153	232	76 (84)	80 (85.5)	82 (87)	84 (88.4)	86 (90.7)	88 (91.9)	91.5 (94.3)	93 (95.5)	95 (97.3)	96 (98.3)
*Iran-regional *			*84.5 *	*85.2 *	*86.5 *	*88 *	*90.5 *	*91.5 *	*94.3 *	*95.4 *	*97.5 *	*99.7 *
36	155	192	81.7 (87.9)	84 (89.1)	87 (91.9)	88 (92.2)	92 (95.1)	93 (96.1)	96.6 (99)	97.58 (99.9)	101.3 (102.2)	101 (103.1)
*Iran-regional *			*87 *	*88.8 *	*90.2 *	*92 *	*95 *	*96 *	*100 *	*101 *	*104 *	*105 *
42	98	147	86.9 (91.4)	88.5 (92.4)	93 (94.7)	96.5 (99)	91 (98.4)	101.3 (101.6)	102 (104)	102 (104)	105 (106.7)	106.3 (107.3)
*Iran-regional *			*91.2 *	*92.5 *	*94.5 *	*96 *	*99.2 *	*100 *	*103.5 *	*104.5 *	*108 *	*108 *
48	80	114	87.2 (94.6)	94.7 (95.4)	95 (98.3)	98 (99)	100 (102.7)	102 (103.3)	105 (107.2)	106 (107.7)	112 (110.8)	110 (111.2)
*Iran-regional *			*93.5 *	*95.8 *	*98 *	*100 *	*103 *	*104 *	*108 *	*109 *	*113 *	*115 *
54	52	57	92.6 (97.6)	94.7 (89.4)	101 (101.5)	100 (102.1)	105 (106.2)	106 (106.7)	110 (110.9)	112 (111.2)	115.8 (114.7)	116.8 (115)
*Iran-regional *			*97.1 *	*100 *	*101 *	*103 *	*106.5 *	*108 *	*111 *	*111.7 *	*115.5 *	*116.3 *
60	34	46	98.3 (100.5)	98.4 (101.2)	103 (104.5)	105 (105.2)	110 (109.4)	110 (110)	112.5 (114.4)	115 (114.8)	117.5 (118.4)	117.6 (118.7)
*Iran-regional *			*101.2 *	*103.5 *	*105 *	*107 *	*110 *	*112 *	*115 *	*116 *	*119.5 *	*119.4 *

*Sample sizes for Iranian normal boys and girls, in all age groups, were 282 and 270 respectively.

**Table 3:** Percentiles of Head Circumference (cm) for age (months) for CH patients and Iranian normal girls and boys (highlighted in italic) and WHO corresponding values (presented in parentheses).

			Percentiles									
Age	Gender		3		15		50		85		97	
(months)	(*N*)		(WHO)		(WHO)		(WHO)		(WHO)		(WHO)	
	Female	Male	Female	Male	Female	Male	Female	Male	Female	Male	Female	Male
3	300	365	33 (37.3)	33 (38.3)	34 (38.3)	34.6 (39.3)	36 (39.5)	36.7 (40.5)	38 (40.8)	39 (41.7)	39 (41.9)	41 (42.7)
Iran-regional *			*37 *	*38 *	*38 *	*39.1 *	*39.5 *	*40.5 *	*40.5 *	*41.5 *	*41.5 *	*42.6 *
6	385	343	38 (40)	39 (41)	39 (41)	40 (43.1)	41 (42.5)	42 (43.3)	43 (43.5)	43.2 (44.6)	44 (44.8)	45 (45.6)
*Iran-regional *			*39.5 *	*41.1 *	*41 *	*42 *	*42.5 *	*43.5 *	*43.5 *	*45 *	*44.8 *	*45.6 *
9	365	311	40 (41.3)	41 (43.6)	42 (43.4)	42 (43.7)	43 (43.8)	44 (45)	45 (45.3)	46 (46.3)	46 (46.3)	47 (47.4)
*Iran-regional *			*41.5 *	*43 *	*43 *	*44 *	*44 *	*45 *	*45 *	*46.5 *	*46.7 *	*47.5 *
12	230	280	43 (43.3)	43 (43.6)	43 (43.6)	44 (44.7)	45 (44.6)	46 (46.1)	46 (46.3)	47.5 (47.4)	48 (47.5)	49 (48.5)
*Iran-regional *			*43 *	*44 *	*44 *	*45 *	*45.3 *	*46.5 *	*46.5 *	*48 *	*47.8 *	*48.9 *
18	241	293	43 (43.6)	44 (44.6)	44 (44.8)	45.5 (46)	46 (46.3)	47 (47.4)	47.5 (47.7)	49 (48.7)	48.75 (48.8)	50 (49.9)
*Iran-regional *			*44.5 *	*45.5 *	*45.5 *	*46.7 *	*47 *	*48 *	*48 *	*49.5 *	*49.5 *	*50 *
24	203	263	44 (44.6)	45 (45.7)	45.8 (45.7)	47 (46.8)	47 (47.2)	49 (48.3)	49 (48.6)	50 (49.7)	50 (49.8)	51 (50.8)
30	149	222	45 (45.3)	45.7 (46.3)	46.7 (46.5)	48 (47.5)	48 (47.6)	49 (48.6)	49 (49.4)	50 (50.4)	50 (50.6)	53 (51.6)
36	153	177	45.8 (45.6)	46.4 (46.8)	47 (47)	48 (48)	49 (48.5)	50 (49.5)	50 (50)	51 (50.9)	52 (51.3)	53 (53.1)

*Sample size for Iranian normal boys (282) and girls (270) in all age groups is the same.

## References

[1] Bain P, Toublanc JE (2002). Adult height in congenital hypothyroidism: prognostic factors and the importance of compliance with treatment. *Hormone Research in Paediatrics*.

[2] Rastogi MV, LaFranchi SH (2010). Congenital hypothyroidism. *Orphanet Journal of Rare Diseases*.

[3] Rose SR, Brown RS, Foley T (2006). Update of newborn screening and therapy for congenital hypothyroidism. *Pediatrics*.

[4] Rosenthal M, Addison GM, Price DA (1988). Congenital hypothyroidism: increased incidence in Asian families. *Archives of Disease in Childhood*.

[5] Hashemipour M, Hovsepian S, Kelishadi R (2009). Permanent and transient congenital hypothyroidism in Isfahan-Iran. *Journal of Medical Screening*.

[6] Haghshenas M, Zahed Pasha Y, Ahmadpour-Kacho M, Ghazanfari S (2012). Prevalence of permanent and transient congenital hypothy-roidism in Babol City-Iran. *Medicinski Glasnik*.

[7] Hashemipour M, Amini M, Iranpour R (2004). Prevalence of congenital hypothyroidism in Isfahan, Iran: results of a survey on 20,000 neonates. *Hormone Research*.

[8] Olney RS, Grosse SD, Vogt RF (2010). Prevalence of congenital hypothyroidism—current trends and future directions: workshop summary. *Pediatrics*.

[9] Kempers MJE, Van Der Sluijs Veer L, Nijhuis-Van Der Sanden RWG (2007). Neonatal screening for congenital hypothyroidism in The Netherlands: cognitive and motor outcome at 10 years of age. *Journal of Clinical Endocrinology and Metabolism*.

[10] Leonardi D, Polizzotti N, Carta A (2008). Longitudinal study of thyroid function in children with mild hyperthyrotropinemia at neonatal screening for congenital hypothyroidism. *Journal of Clinical Endocrinology and Metabolism*.

[11] Hashemipour M, Hovsepian S, Kelishadi R (2009). Permanent and transient congenital hypothyroidism in Isfahan-Iran. *Journal of Medical Screening*.

[12] Rose SR, Brown RS, Foley T (2006). Update of newborn screening and therapy for congenital hypothyroidism. *Pediatrics*.

[13] Sabri MR, Shahriari H, Hashemipour M (2006). Congenital cardiac malformations in congenital hypothyroid patients in Isfahan. *Journal of Research in Medical Sciences*.

[14] Hashemipour M, Nasri P, Hovsepian S (2010). Urine and milk iodine concentrations in healthy and congenitally hypothyroid neonates and their mothers. *Endokrynologia Polska*.

[15] Hashemipour M, Amini M, Talaie M (2007). Parental consanguinity among parents of neonates with congenital hypothyroidism in Isfahan. *Eastern Mediterranean Health Journal*.

[16] Adibi A, Haghighi M, Hosseini SR, Hashemipour M, Amini M, Hovsepian S (2008). Thyroid abnormalities among first-degree relatives of children with congenital hypothyroidism: an ultrasound survey. *Hormone Research*.

[17] Mahjoubi F, Mohammadi MM, Montazeri M, Aminii M, Hashemipour M (2010). Mutations in the gene encoding paired box domain (PAX8) are not a frequent cause of congenital hypothyroidism (CH) in Iranian patients with thyroid dysgenesis. *Arquivos Brasileiros de Endocrinologia e Metabologia*.

[18] Bongers-Schokking JJ, Koot HM, Wiersma D, Verkerk PH, De Muinck Keizer-Schrama SMPF (2000). Influence of timing and dose of thyroid hormone replacement on development in infants with congenital hypothyroidism. *Journal of Pediatrics*.

[19] NECH Collaborative (1985). Neonatal hypothyroidism screening: status of patients at 6 years of age. *Journal of Pediatrics*.

[20] Grant DB (1994). Growth in early treated congenital hypothyroidism. *Archives of Disease in Childhood*.

[21] Fisher DA, Sperling MA (1996). Disorders of the thyroid in the newborn and infant. *Pediatric Endocrinology*.

[22] (2003). Screening for congenital hypothyroidism. *Thyroid*.

[23] Klein RZ, Mitchell MH, Braverman LE, Utiger RD (2000). Hypothyroidism in infants and children. *The Thyroid*.

[24] Frank JE, Faix JE, Hermos RJ (1996). Thyroid function in very low birth weight infants: effects on neonatal hypothyroidism screening. *Journal of Pediatrics*.

[25] de Onis M, Woynarowska B (2010). WHO child growth standards for children 0-5 years and the possibility of their implementation in Poland. *Medycyna Wieku Rozwojowego*.

[26] Vazirian S, Sedighnezhad A (2003). Update of growth percentiles for children of an Iranian population. *Archives of Iranian Medicine*.

[27] Kik E, Noczyńska A (2011). Evaluation of physical development of children with congenital hypothyroidism detected in the screening test-personal observations. *Pediatric Endocrinology, Diabetes, and Metabolism*.

[28] Chiesa A, De Papendieck LG, Keselman A, Heinrich JJ, Bergada C (1994). Growth follow-up in 100 children with congenital hypothyroidism before and during treatment. *Journal of Pediatric Endocrinology*.

[29] Moschini L, Costa P, Marinelli E (1986). Longitudinal assessment of children with congenital hypothyroidism detected by neonatal screening. *Helvetica Paediatrica Acta*.

[30] Bucher H, Prader A, Illig R (1985). Head circumference, height, bone age and weight in 103 children with congenital hypothyroidism before and during thyroid hormone replacement. *Helvetica Paediatrica Acta*.

[31] Sato H, Sasaki N, Aoki K, Kuroda Y, Kato T (2007). Growth of patients with congenital hypothyroidism detected by neonatal screening in Japan. *Pediatrics International*.

[32] Sun Q, Chen YL, Yu ZB (2012). Long-term consequences of the early treatment of children with congenital hypothyroidism detected by neonatal screening in Nanjing, China: a 12-year follow-up study. *Journal of Tropical Pediatrics*.

[33] Agulló AG, Vicens-Calvet E, Lezcano AC, Esteve MB, Vilalta NP (2010). Growth and maturation in the patients with congenital hypothyroidism detected by the neonatal screening program in Catalonia, Spain (1986–1997). *Medicina Clínica*.

[34] Adachi M, Asakura Y, Tachibana K (2003). Final height and pubertal growth in Japanese patients with congenital hypothyroidism detected by neonatal screening. *Acta Paediatrica*.

[35] Morin A, Guimarey L, Apezteguía M, Ansaldi M, Santucci Z (2002). Linear growth in children with congenital hypothyroidism detected by neonatal screening and treated early: a longitudinal study. *Journal of Pediatric Endocrinology and Metabolism*.

[36] Siragusa V, Terenghi A, Rondanini GF (1996). Congenital hypothyroidism: auxological retrospective study during the first six years of age. *Journal of Endocrinological Investigation*.

[37] Heyerdahl S, Ilicki A, Karlberg J, Kase BF, Larsson A (1997). Linear growth in early treated children with congenital hypothyroidism. *Acta Paediatrica*.

[38] Salerno M, Micillo M, Di Maio S (2001). Longitudinal growth, sexual maturation and final height in patients with congenital hypothyroidism detected by neonatal screening. *European Journal of Endocrinology*.

[39] Ng SM, Wong SC, Didi M (2004). Head circumference and linear growth during the first 3 years in treated congenital hypothyroidism in relation to aetiology and initial biochemical severity. *Clinical Endocrinology*.

[40] Hashemipour M, Hovsepian S, Kelishadi R (2012). High prevalence of congenital hypothyroidism in Isfahan: do familial components have a role?. *Advanced Biomedical Research*.

